# Taxonomical loss for weed seedlings image classification

**DOI:** 10.1038/s41598-025-33961-0

**Published:** 2026-01-24

**Authors:** Hans-Olivier Fontaine, Samuel Foucher, Edith Fallon, Marie-Josée Simard, Etienne Lord

**Affiliations:** 1 Department of Applied Geomatics, Université de Sherbrooke, 2500 boulevard de l’Université, Sherbrooke, QC J1K 2R1 Canada; 2 Agriculture and Agri-Food Canada, St-Jean-sur-Richelieu Research and Development Centre, 430 Gouin Boulevard, Saint-Jean-sur-Richelieu, QC J3B 3E6 Canada

**Keywords:** Few-shot learning, Taxonomy, Weed classification, Phenology, Deep learning, Computational biology and bioinformatics, Ecology, Ecology, Plant sciences

## Abstract

Accurate classification of weed seedlings is a key challenge to address in order to advance precision weed management and reduce pesticide use. In this study, an original image dataset, the Weed Phenological Dataset (WPD), with annotated early growth stages is introduced. Furthermore, a novel deep learning taxonomic loss function for few-shot learning was evaluated. Using hierarchical structures to direct the classification process, this taxonomic loss function introduces dynamic margins during the computation. In experiments using the taxonomic loss using the ResNet-50 architectures across different plant image datasets, this taxonomic approach allowed better clustering according to the Silhouette scores when compared with triplet loss using 100 images per class. It also led to better identification of weed seedlings at early growth stages. Although the new taxonomic loss did not consistency improve classification results for all the deep learning model architectures, it open new research avenues for the robotization of agriculture.

**Supplementary Information:**

The online version contains supplementary material available at 10.1038/s41598-025-33961-0.

## Introduction

Image classification algorithms are becoming increasingly important for weed management automation. For example, the automatic classification of crops and non-crop plant species is necessary for the targeted application of herbicides to control weeds, allowing a reduction of their associated environmental impacts^1^. Lack of proper weed control can result in significant yield losses, typically starting around 20%, unless the crop is transplanted^2^, and potentially reaching 100% in horticultural crops that are not competitive, such as carrots^3,4^. In order to properly manage weeds using either physical, mechanical, biological or chemical options, they need to be identified early, at low phenological stages since small weeds are easier to control^5,6^. Mechanical weeding illustrates this point well: it only works effectively on small weeds with fewer than four leaves, and some tools are only efficient when weeds have barely sprouted^7^. Herbicide directives are generally less stringent but also recommend applications on small and actively growing annual weeds^8^. Examples of recommended leaf stages for different herbicides include: fewer than 2 leaves for imazethapyr^9^, less than 4 leaves for bromoxynil^10^ and, 2 to 5 leaves for fluazifop-p-butyl and clethodim^11,12^.

Currently, there is an increasing trend to replace traditional weed identification using simple morphological features, such as the presence or absence of trichomes, with algorithms based on machine learning or deep learning methods^13^. Precise weed identification allows the creation of weed maps that can improve precision farming^14^. Different deep learning model architectures can be trained to classify plant images^15,16^. Convolutional Neural Network (CNN) architectures, such as Residual Network (ResNet)-based models, are among the most frequently utilized^17,18^. However, these models generally require more computer memory, limiting their direct application in embedded devices such as smart cameras. Lightweight models such as MobileNets^19^, with their simplified depth-wise separable convolutions, linear bottlenecks, inverted residual structures and lightweight attention modules, are preferred for embedded platforms^20,21^. Although, MobileNet models generally perform with 5–10% (V1) or 3–6% (V2) less precision than their counterparts^16,22^, they operate at faster speeds due to the reduced number of calculations.

Supervised training of deep learning models requires a high volume of sample images, often in the thousands, to accurately identify plants or other subjects in various environments^15^. This requirement is particularly challenging when weed seedlings need to be classified since they are typically underrepresented or absent in most image datasets^23,24^. Traditionally, the precise identification of weed species requires expert knowledge and experience^25^, and is complexified by the morphological similarity of seedlings during early developmental stages, when they only have 1 to 4 true leaves (BBCH stages 10–14)^26^.

Recent advances in Few-Shot Learning (FSL) techniques have demonstrated that these deep learning methods can differentiate classes using small image datasets^27–29^. Some of these techniques use contrastive learning such as Siamese networks^30^, that learn to associate positive images according to their characteristics, while learning the discriminative characteristics of other classes. This is achieved through an encoder that transforms sets of images into learned embedding (vectors), which are then compared using a similarity or loss function. However, the standard contrastive learning approaches may struggle to capture the hierarchical relationships between plant species (e.g., species within the same genus or family), which are crucial for accurate weed identification.

This project was thus initiated to extend the work of Ge^31^ and hierarchical triplet loss (HTL). The HTL loss function employs a dynamic distance matrix construction and an adaptive violation margin during deep neural network training, allowing the networks to learn subtle differences in image characteristics, similar to the way weed scientists classify plant species. However, in Ge’s work, the hierarchical structure is learned during the training process. It was hypothesized that this approach, when given a defined taxonomic tree, would help the deep learning model identify related weed species (in the same genus or family) at early growth stages better than other approaches when using a low number of annotated images for each species.

Henceforth, the contributions of this paper are as follows:

1. The description of a new annotated weed seedling dataset at low phenological stages (BBCH 10–14) with related species;
2. The use of a defined taxonomy or classification key, known beforehand, to direct the supervised learning process.
3. The inclusion of a new taxonomic loss with dynamic margin function, which allows the embedding feature vectors to be aligned more precisely with taxonomic tree hierarchy;

The different contributions were demonstrated using deep learning networks with either a MobileNet or ResNet-50 backbone, without implementing any image augmentation techniques.

## Materials and methods

### Weed phenological dataset

**Fig. 1:**
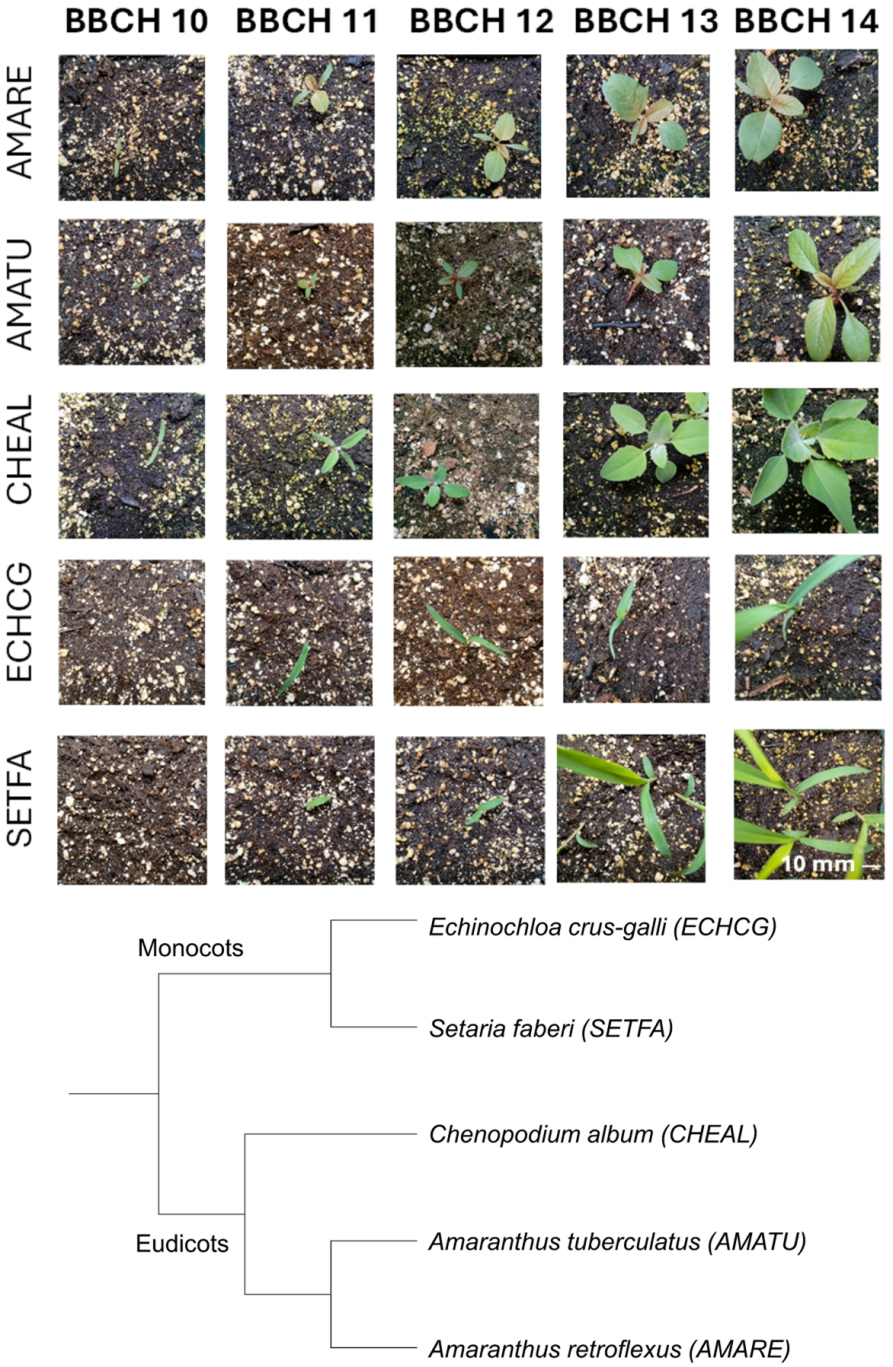
Overview of the Weed Phenological Dataset image and taxonomy. Final image dimensions are 512 × 512 pixels.

A novel image dataset of five weed species (Weed Phenological Dataset; WPD) containing 3,920 images (Table 1) with very different [grass like (monocotyledonous) vs. broadleaved (dicotyledonous) weeds] and very similar (species of the same genus) morphological characteristics were created (Fig. 1). A total of 100 plants of each species were grown in a greenhouse located at Agriculture and Agri-Food Canada facilities (Saint-Jean-sur-Richelieu, Québec, Canada), from seeds harvested on AAFC experimental farms, on a minimum of five individual plants. Seeds were kept at 5 °C until planted. Plants were grown at a temperature of 25/18°C, day/night, and a 16/8 h photoperiod in 100 mm diameter pots filled with a potting soil mix composed of sphagnum peat moss (60–70%) and other ingredients (ProMix^®^ BX M, Premier Tech). They were fertilized with a nutrient solution (N, P_2_O_5_, K_2_O; 20-20-20 Plant-Prod^®^, 1 g L^− 1^, 300 ppm) once a week. Photographs were taken up to the BBCH 14 stage, which took 21 days, using a DELL UltraSharp 4 K camera (3280 × 2160 pixels resolution), with illumination provided by a Godox M1 lamp, at a constant height (750 mm), resulting in an image of 100 × 100 mm (2160 × 2160 px, ~ 0.5 mm/pixel resolution) after cropping. Photographs were then resized to 512 × 512 pixels for the different model training and simulations.

All the selected weed species were either common in row crops in North America or were problematic due to either, their exotic origin from Asia (*Setaria faberi)* or the presence of biotypes with multiple herbicide resistance (*Amaranthus tuberculatus*)^32,33^. In contrast to other plant image datasets^25^, the dataset includes images with manually annotated and verified growth stages. The annotated images (BBCH 10 to 14) corresponding to plants with cotyledons or unexpanded leaves up until at least four expanded leaves were present^26^, represent a total of 3,230 images.

The selected species included grasses such as Barnyard grass (*Echinochloa crus-galli* (L.) Beauv., ECHCG) and Giant foxtail (*Setaria faberi* Herm., SETFA), belonging to the monocotyledons, and visually distinguishable from dicotyledonous species such as Lamb’s-quarters (*Chenopodium album* L., CHEAL), Redroot pigweed (*Amaranthus retroflexus* L., AMARE) and Waterhemp (*Amaranthus tuberculatus* (Moq.) Sauer var. rudis (Sauer), AMATU) by the presence of slender parallel-veined leaves^34^. Among the grasses, Barnyard grass has a flat cross section i.e. stems are flattened, especially near the base, no ligule (this is not visible without manipulating the plant), and no hairs^35^. Giant foxtail leaves have hairs, the ligule consists of a row of fine hairs (again not visible without spreading leaves apart) and the cross section is not flat but oval to round^36^. As for the broad-leaved dicotyledonous species, lamb’s-quarters leaves are mealy (appear covered by a white powder) and the cotyledons have no midvein^36,37^. Both *Amaranthus* species have cotyledons with mid veins. Amaranthus species are not easy to distinguish when seedlings are small. Redroot pigweed plants can be distinguished from other *Amaranthus* species by the presence of many hairs on leaf margins and especially on the stem from the third leaf stage onwards^37^. Compared to waterhemp, the first leaves are round to oval while waterhemp leaves are lance-shaped^36^. The taxonomy used for the classification reflected the current species phylogeny (Fig. 1), with the distance set as 1.0 between each taxonomical level.

**Table 1:** Summary of plant seedling datasets.

Name	Images^1^	Plant species and images^2^
WPD	3920 3230 (BBCH10-14) 2467 (BBCH10-12)	**5 weed species**^**3**^ **(BBCH09 to BBCH21+)** *Amaranthus retroflexus* (AMARE): 934 / 658 / 570 *Amaranthus tuberculatus* (AMATU): 409 / 292 / 238 *Chenopodium album* (CHEAL): 832 / 655 / 498 *Echinochloa crus-galli* (ECHCG): 768 / 725 / 581 *Setaria faberi* (SETFA): 977 / 900 / 580
DeepWeeds^38^	8403	**8 invasive weed species**: *Chromolaena odorata*: 1,074 *Cryptostegia grandiflora*: 1,009 *Lantana camara*: 1,063 *Parkinsonia aculeata*: 1,031 *Parthenium hysterophorus*: 1,022 *Stachytarpheta indica*: 1,016 *Vachellia nilotica* subsp. indica: 1,062 *Ziziphus mauritiana*: 1,126
Plant seedlings^21^	5539	**12 plant species (weeds & crops)**: *Alopecurus myosuroides*: 309 *Apera spica-venti*: 762 *Beta vulgaris*: 463 *Capsella bursa-pastoris*: 274 *Chenopodium album*: 538 *Galium aparine*: 335 *Geranium pusillum*: 576 *Sinapis arvensis*: 452 Stellaria media: 713 *Tripleurospermum inodorum*: 607 *Triticum aestivum*: 253 *Zea mays*: 257

^1^Total images used in the simulations. ^2^Total images for each plant species. ^3^Image counts for the whole dataset, BBCH10 to 14 or BBCH10-12 respectively.

### Other weed datasets

The DeepWeeds dataset^38^ was used in our experiment as it presented weed images taken under different environmental conditions across Australian pastures (Supplementary Fig. 1). The photographs also featured species that were not targeted by this study. The whole dataset comprises 17,509 images spanning 8 weed species and negative images (soil background), aiming to facilitate deep learning research in agriculture. For the simulations, images were resized at a resolution of 224 × 224 pixels, as used by the DeepWeeds dataset authors, and the negative images were not used during the training of the models.

The Plant Seedlings dataset^21^ is another weed science oriented datasets. It comprises 12 species (~ 5,500 images) with ten weed species and two crop species (*Zea mays* L. and *Triticum aestivum* L.) (Supplementary Fig. 2). This dataset images were captured in greenhouse conditions with good illumination. Image sizes within this dataset is variable, but the resolution is set to 10 pixels per mm. For this study, individual weed seedlings were isolated from the full images and resized to 64 × 64 pixels before the training steps. Finally, the hierarchical trees used were based on the species phylogeny^39^, with the distance set as 1.0 between each taxonomical level.

### New taxonomical loss function

**Fig. 2:**
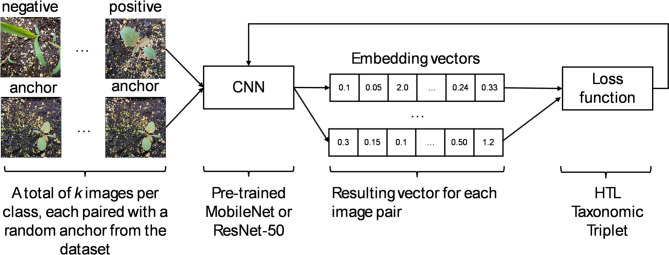
Schematic view of the experiment deep learning networks. The positive and negative images are classified by the selected convolutional neural network (CNN) in relation to a reference image (anchor). During the training steps, their final embedding vectors distances are evaluated by the loss function. In this study, the loss function was the classical triplet loss, the hierarchical triplet loss (HTL) or a taxonomic loss function.

Contrastive learning is designed to learn representations by maximizing similarity between similar (positive) sample pairs while minimizing similarity with dissimilar (negative) samples (Fig. 2). During training, the latent space representations are encoded in feature vectors, while being refined through forward propagation and iterative optimization^40^. The loss function explicitly models the sample relationships and therefore evaluates the model’s ability to map similar vectors for sets of similar classes as well as dissimilar vectors for sets of different classes. Different loss functions, including contrastive loss^40^, triplet loss^41^, quadruplet loss^42^, and hierarchical triplet loss (HTL)^31^ have been created for different image classification applications.

Commonly, the calculation of the standard triplet loss function, where geometric distances correspond to semantic relationships, focuses on minimizing the distance between positive pairs and maximizing the distance between negative pairs, which can lead to an inefficient use of the training data. A significant limitation is the dependence on fixed margin values to separate positive and negative pairs, as this approach may fail to generalize across varying data distributions and complexities^43,44^. Additionally, the standard triplet loss model usually requires a careful balancing of positive and negative samples, and thus is sensitive to pair selection. Hierarchical triplet loss (HTL)^31^ enhances the approach by organizing training samples based on a hierarchical class structure, generating a non-random selection of the samples reducing redundancy. This strategic sampling contributes to faster model convergence and heightened performance across various tasks^31^. Indeed, efficient sampling strategies become crucial when handling plant species that are morphologically close, as images from the same plant family or genus present some overlapping feature similarities at different phenological stages^45^. This a hierarchical class strategy replicates methods used by weed scientists and agronomists such as custom keys, identifying subtle differences in leaf shape, texture, color or venation^46^, during early phenological stages instead of classical botanical keys generally based on flower and fruit characteristics to accurately identify weed seedlings^47^.

The general triplet loss function used in different FSL methods such as Siamese networks^30^, can be described as follows. Given a triplet of image samples consisting of an anchor image (*a*), a positive image sample (*p*), and a negative image sample (*n*), let:

- *x*_*a*_ ∈ ℝᵈ be the *d*-dimensional embedding vector of the anchor sample.
- *x*_*p*_ ∈ ℝᵈ be the *d*-dimensional embedding vector of the positive sample.
- *x*_*n*_ ∈ ℝᵈ be the *d*-dimensional embedding vector of the negative sample.

Where *d* is the length of the embedding vector. The triplet loss function *L*_*triplet*_ is then defined as (Eq. 1):

1 $$\:{L}_{triplet}=max(0,\:dist({x}_{a},{x}_{p})-dist({x}_{a},{x}_{n})+m).$$

where:

- *dist*(·,·) denotes the distance metric between two embedding vectors.
- *m* > 0 is the margin parameter that enforces a minimum separation between the positive and negative pairs.

This loss function aims to ensure that the distance between the anchor and positive sample *dist*(*x*_*a*_, *x*_*p*_) is smaller than the distance between the anchor and negative sample *dist*(*x*_*a*_, *x*_*n*_) by at least a margin *m*. When this condition is satisfied, the loss becomes zero. The standard triplet loss effectively pulls positive pairs closer together in the embedding space while pushing negative pairs apart, leading to embedding that capture semantic similarity between samples. However, if a priori knowledge about sample is known, such when a known hierarchy is used, the *m* parameter could be replaced by a dynamic margin. Ge and collaborators^31^ used the following margin parameter *m*_*a*_ (Eq. 2) to classify weed photographs:

2 $$\frac{ab}{cd}{ab}{\:{m}_{a}=\beta\:+{d}_{H\left({T}_{a},{T}_{n}\right)}-{S}_{a}}.$$

Where $\:\beta\:$ was a constant ($\:\beta\:$ =0.1), $\:{d}_{H\left({T}_{a},{T}_{n}\right)}$ was the distance between the merged levels of the anchor class images and the negative class images, and $\:{S}_{a}$ was the average distance between samples in the anchor class. This improvement of the triplet loss generated a better distribution of anchor images by pushing away images with different semantics even when they were from the same class.

In this study, a new taxonomical loss formulation was explored considering the positive and negative samples. The margin parameter *m* was replaced by the difference in the taxonomical distance *m*_*t*_ calculated between the anchor and the positive and negative samples such that (Eq. 3):

3 $$\:{L}_{taxonomic}=\mathrm{m}\mathrm{a}\mathrm{x}(0,\:dist({x}_{a},{x}_{p})-dist({x}_{a},{x}_{n})+{m}_{t}),$$

with :

4 $$\:{m}_{t}=N\left({T}_{p}-{T}_{n}\right).$$

Where in Eqs. 3 and 4 the different terms are:

- $\:\:dist\left({x}_{a},{x}_{p}\right)\:$is the embedding vector distance between the anchor and the positive sample.
- $\:\:dist\left({x}_{a},{x}_{n}\right)\:$is the embedding vector distance between the anchor and the negative sample.
- $\:{T}_{p}$ ∈ ℝ is the taxonomic distance between the anchor and the positive sample.
- $\:{T}_{n}$∈ ℝ is the taxonomic distance between the anchor and the negative sample.

And *N*(·) is a min-max normalization function defined as (Eq. 5):

5 $$N(z) = (z - min(z)) / (max(z) - min(z) + \epsilon)$$

The factor ε = 10⁻⁶ is a small constant to prevent division by zero and the Euclidean distance was used for the distance between the embedding vectors. The normalization ensures the *m*_*t*_ term is bounded between 0 and 1. A greater value of *m*_*t*_ allows classes to be placed further apart in the feature space created by the neural network. Embedding generated from two different image classes would require a separation distance of at least *m*_*t*_ to get a loss of zero. A loss of zero would therefore indicate that the distance between the negative embedding and the anchor embedding would have to be at least equal to the distance between the positive embedding and the anchor embedding, plus the margin *m*_*t*_. Having this dynamic taxonomical margin thus ensure that the resulting embedding follows the provided tree topology.

### Deep learning training and simulations

Convolutional neural networks in a configuration, similar to the work of Wang and Wang^29^, were used in this study (Fig. 1), with three different loss functions: hierarchical triplet loss^31^, taxonomic or standard triplet loss. No image augmentation techniques^48^ were used during the training steps in order to highlight the differences in loss function on the resulting embedding vectors, and to ensure that there were no biases generated by unequal augmentation between each simulation.

The different simulation codes and models were implemented using Pytorch (v2.2.0) with CUDA support in Python 3.9. For the baseline network (referred as general), neural networks available in Pytorch such as Resnet-50 (ResNet)^18^ with pretrained weights (ImageNet1K_v1), or MobileNet (V3)^22^ with pretrained weights (Small weight, imageNet1K-V1), were used independently. The same pretrained networks were also used as the backbone of the Siamese networks. Taxonomical tree manipulation was carried out using the Biopython library(v1.83) with networkx (v3.2) to import the tree structures and to calculate the resulting distance matrix using the Dijkstra algorithm.

Training and test simulations were carried out ten times (*N* = 10), with different staring values of random seeds. Batch sizes of 64 using the novel WPD dataset (Sect. 3.1), or 128 for the other datasets with their reduced image sizes, were used. A final vector embedding size of 64 was used for all loss function simulations, along a dynamic learning rate of 0.001 with a Reducer on Plateau (patience = 20, threshold = 0.01, factor = 0.1). For each simulation replicate, the images for each class were randomly separated prior to the training phase. A specific number of images for each class (*k*) was used for training (training set) in the ranges 5 to 200, with the exception that for the specific BBCH growth stage models (Sect. 3.3), only 10 images for each class (*k* = 10) were used. The remaining images in each class were distributed evenly (50/50%) into validation and test sets up to 250 for each set. The different models (ResNet-50^49^ or MobileNet^21^ and loss functions (general, HTL, taxonomic or triplet) were evaluated on the same data for each replicate. For HTL loss, class hierarchy was re-evaluated every 5 epochs with a maximum of 16 levels.

While studies using FSL techniques use relatively high number of iterations (200 up to 30 K)^28–30,38^ with different training strategies and goals, the selected maximum number of epochs was based on the good separation of individual clusters as visualized with a t-SNE clustering^50^ of the final embedding vector. A total between 50 and 100 epochs were used for the experiments and the model with the smallest training loss was evaluated for each replicate. Individual class assignment was performed by calculating the Euclidean distance between the final image embedding to the nearest embedding vector obtained from the training set. Simulations and training were carried out on a workstation with a NVIDIA RTX 8000 GPU (48 Gb VRAM) on a dual Intel Xeon Gold (5120) system equipped with 240 Gb of RAM.

Results including precision, recall, F1-score, and Silhouette score metrics were calculated using the scikit-learn python package (v0.24.2). Silhouette scores^51^ were calculated for each vector embedding to evaluate the capacity of each loss function to regroup images belonging to a similar class, indicating better separation. The Silhouette score compares the average intra-cluster distance with the mean distance to the nearest cluster, capturing both how well clustered the points are and how distinct the different cluster are from one another. Higher scores (closer to one) indicate well-defined clusters, where objects are tightly grouped with their cluster neighbors. A negative Silhouette score indicates indiscernible clusters.

For the different experiments, the average (mean) and standard deviations (SD) is reported and the complete results are provided in the supplementary information. Visualization of the different class embedding was carried out using *t*-SNE (scikit-learn) using 2 as the number of components and perplexity, and a fixed random seed for replicability. Non-parametric Wilcoxon rank sum exact tests with Bonferroni *P*-value corrections were used to separate the groups and carried out using R (v.4.3.1). Figures were generated using R or python and code can be found at: https://github.com/etiennelord/TaxonomicalLoss.

A summary of the methods is available in Supplementary Fig. 3.

## Results

### Classification using hierarchical loss

An overview of the WPD dataset is presented in Fig. 2 with the taxonomy used in the simulations. The dataset was designed to have a single species in each image, with some of the image presenting multiple plants (e.g. SETFA, BBCH 14, Fig. 2).

**Fig. 3:**
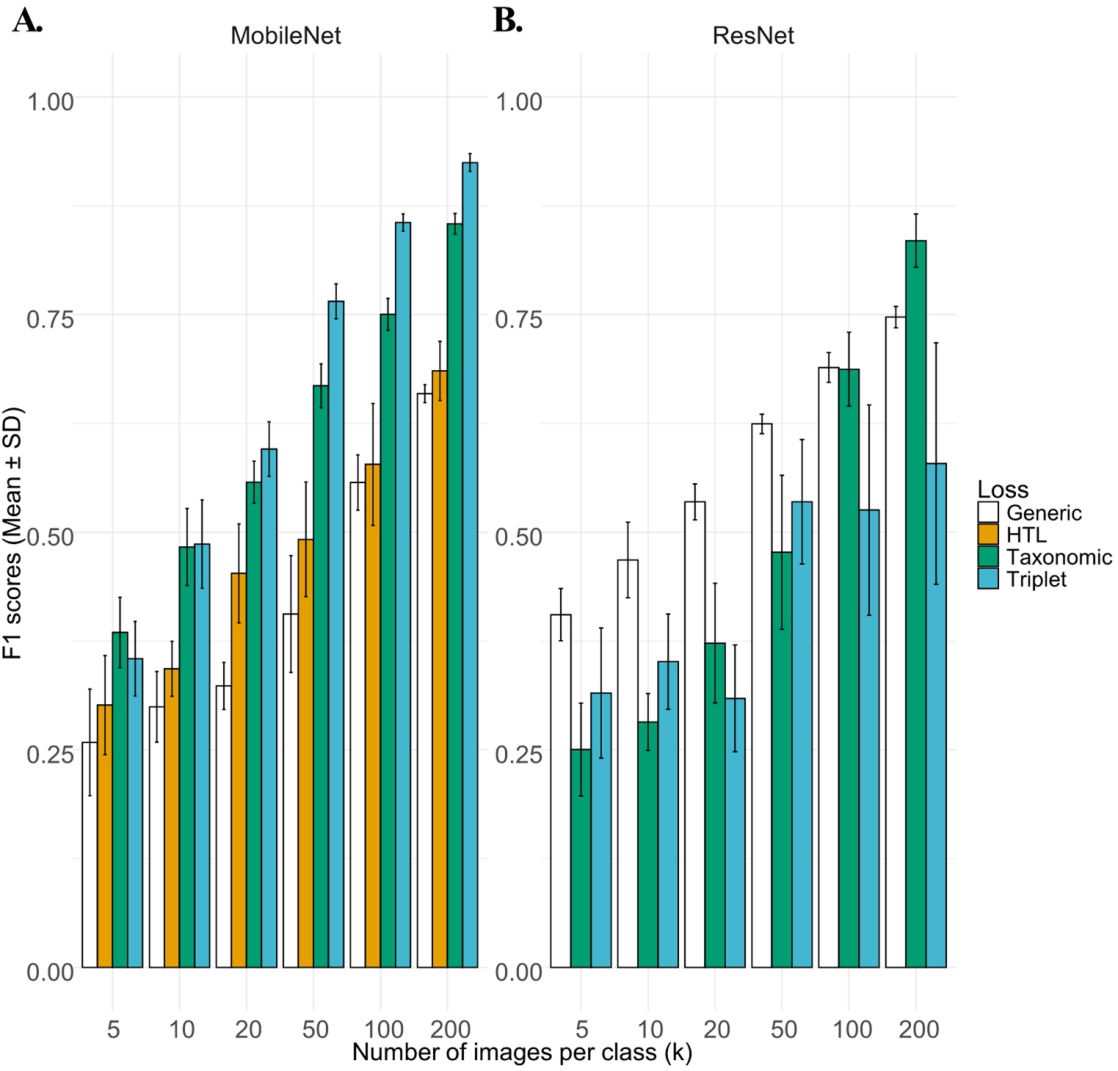
F1 score classification results for the Weed Phenological Dataset using different loss functions using either (**A**) MobileNet or (**B**) ResNet as the base architecture with varying number of images per class (*k*). Simulations were independently carried out (*N* = 10). Generic loss refers to a non-Siamese MobileNet or ResNet model with standard cross entropy loss.

Investigation on hierarchical loss started by using this dataset with our implementation of HTL loss^31^ using MobileNet (Fig. 3A). It was found that between 10 and 50 images for each class, the HTL loss with hierarchical class evaluation and dynamic image selection resulted in small improvements in the F1 score over the generic MobileNet model (Fig. 3 and Supplementary tables, HTL vs. generic, *P* < 0.05). However, this advantage was not significant when 100 or 200 images per class was used. Using a fixed taxonomy was then considered with a novel loss function (Sect. 2.5; Eqs. 3–5). This taxonomic loss was found to significantly improve the classification results of the WPD dataset when compared to both the general CNN models (generic model) and HTL loss (Fig. 3A, MobileNet F1, *P* < 0.01, *k* = 5-200). However, when compared to the standard triplet loss (Fig. 3A), the new taxonomic loss was found to underperform (*P* < 0.01, F1, *k* = 50–200).

**Table 2:** Classification of WPD using ResNet-50.

Loss^1^	k	Precision	Recall	F1	Silhouette^2^
Generic	10	0.49 ± 0.04	0.47 ± 0.04	0.47 ± 0.04	–
Generic	20	0.55 ± 0.02	0.53 ± 0.02	0.53 ± 0.02	–
Generic	50	0.63 ± 0.01	0.62 ± 0.01	0.62 ± 0.01	–
Generic	100	0.70 ± 0.02	0.69 ± 0.02	0.69 ± 0.02	–
Generic	200	0.75 ± 0.01	0.74 ± 0.01	0.75 ± 0.01	–
Taxonomic	10	0.30 ± 0.03	0.29 ± 0.03	0.28 ± 0.03	0.22 ± 0.13
Taxonomic	20	0.40 ± 0.06	0.36 ± 0.06	0.37 ± 0.07	0.11 ± 0.05
Taxonomic	50	0.51 ± 0.09	0.47 ± 0.08	0.48 ± 0.08	0.55 ± 0.05
Taxonomic	100	0.72 ± 0.04	0.68 ± 0.04	0.69 ± 0.04	0.81 ± 0.08
Taxonomic	200	0.85 ± 0.03	0.83 ± 0.03	0.84 ± 0.03	0.80 ± 0.01
Triplet	10	0.40 ± 0.05	0.36 ± 0.05	0.35 ± 0.05	0.23 ± 0.09
Triplet	20	0.32 ± 0.06	0.30 ± 0.06	0.31 ± 0.06	0.05 ± 0.01
Triplet	50	0.57 ± 0.08	0.52 ± 0.06	0.53 ± 0.07	0.34 ± 0.15
Triplet	100	0.55 ± 0.12	0.52 ± 0.11	0.53 ± 0.11	0.28 ± 0.19
Triplet	200	0.60 ± 0.13	0.57 ± 0.13	0.58 ± 0.13	0.22 ± 0.12

^1^Generic loss refers to the original model used in classification with standard cross-entropy loss. ^2^Sillhouette scores of the resulting clusters after applying t-SNE to the training set. Simulations were independently carried out (*N* = 10) and results represent the mean ± SD.

Simulations were also carried out using a ResNet-50 base network (Table 2; Fig. 3B). While the taxonomical loss showed poor performance at low *k* values (*k* = 10,20, 50), it dramatically improves at higher *k* values (Table 2). Best overall performance was obtained at *k* = 200 with a F1 score of 0.8350. Thus, in contrast to the MobileNet architecture, the ResNet network showed improved classification results. With the same number of images per class (*k* = 100), it was however observed that triplet loss showed higher variation in F1 score and lower classification results (*P* < 0.001, ResNet, taxonomic vs. triplet loss, F1 scores of 0. 0.718 ± 0.037 vs. 0.546 ± 0.124).

**Fig. 4:**
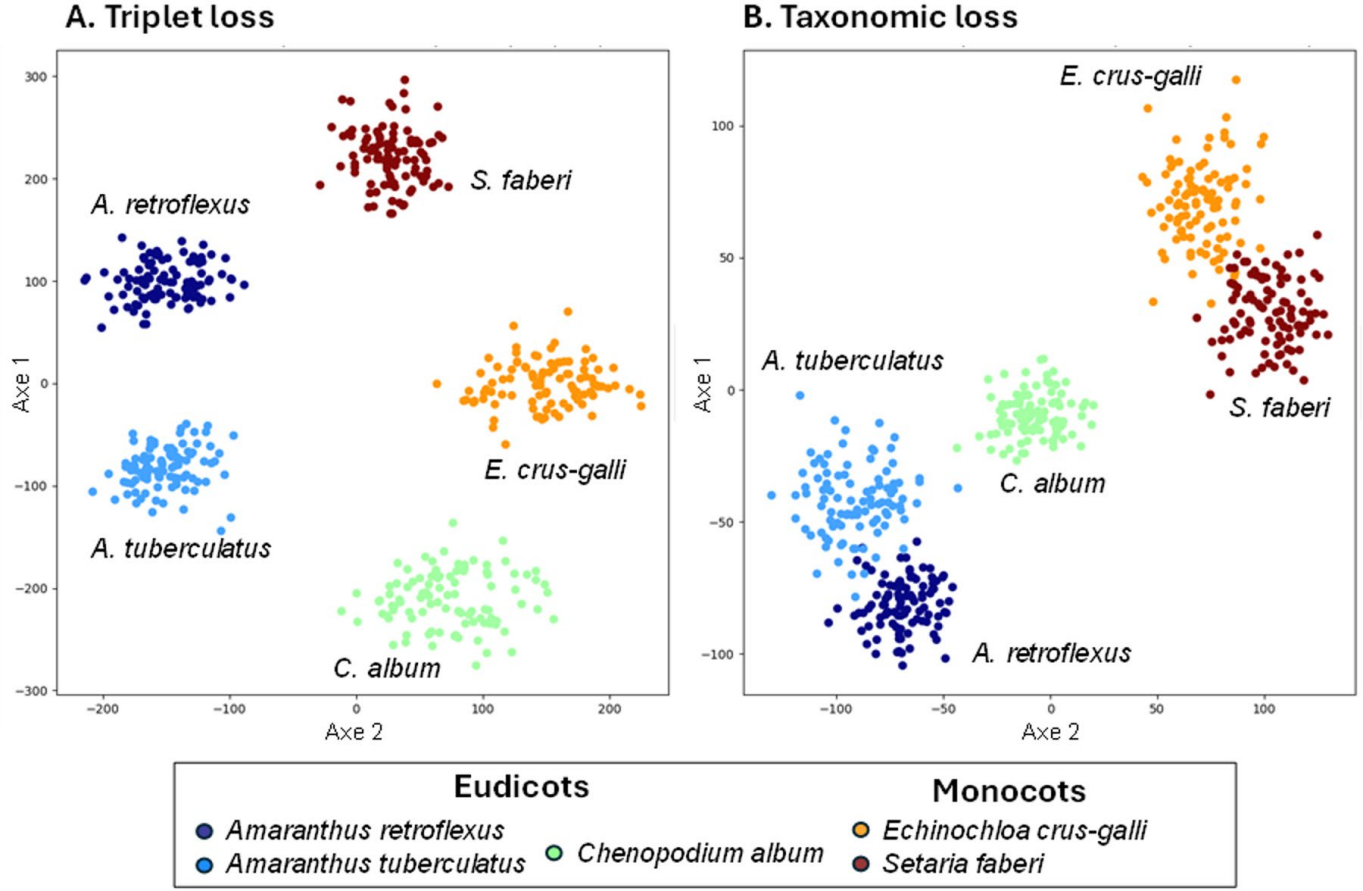
Representative visualization of the WPD training set final embedding vector using *t*-SNE clustering (*k* = 100) for each classes after 50 epochs with ResNet using standard triplet loss (**A**), or using taxonomic loss (**B**) using the same training data.

It was also observed (Fig. 4) that triplet loss clustering of the embedding vector resulted in a more geometric disposition of the different classes (Fig. 4A), without a specific arrangement, while the taxonomic loss followed more closely the provided hierarchical classification (Fig. 4B). Silhouette score between the taxonomical and the triplet loss clustering of the embedding vectors confirmed that the taxonomical loss created more distinct clusters for this model architecture (*P* < 0.001. Taxonomical lost vs. Triplet loss, Silhouette score 0.81 ± 0.08 vs. 0.28 ± 0.19, *k* = 100).

**Fig. 5:**
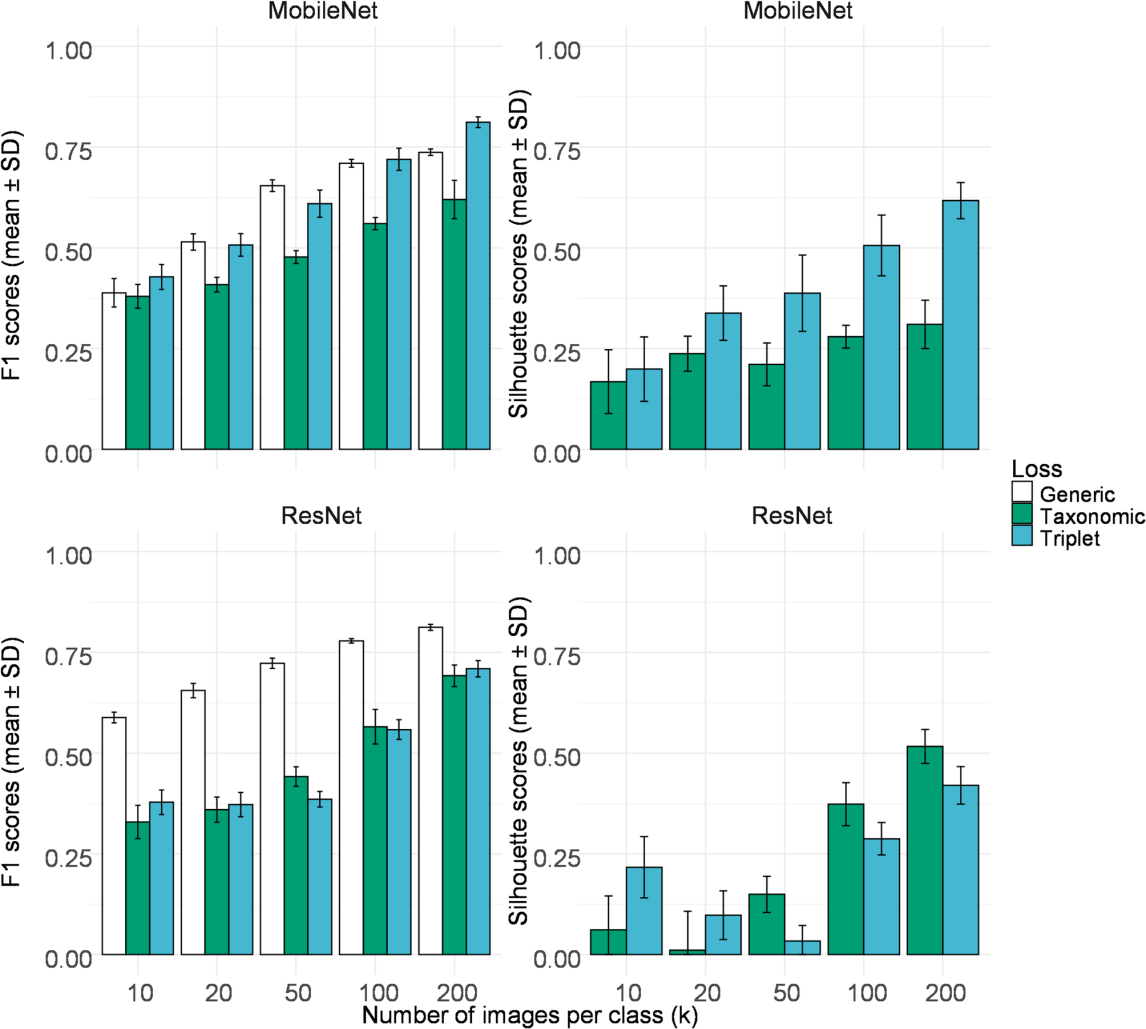
F1 and Silhouette scores classification results for the DeepWeeds datasets using different loss functions with either MobileNet or ResNet as the base architecture with varying number of images per class (*k*). Simulations were independently carried out (*N* = 10).

### Classification of the deepweeds and plant seedlings datasets

Evaluation of the taxonomical loss was carried out on the DeepWeeds and Plant seedlings datasets using the MobileNet and ResNet deep learning architecture. The DeepWeeds dataset presents weed and plant species in their natural environment, while the Plant seedlings dataset was prepared similarly to the WPD dataset, but contains more species (see Supplementary Figs. 1 and 2).

For the DeepWeeds dataset, it was found that the generic network architectures performed better than the taxonomical loss or triplet loss (Fig. 5), triplet loss only surpassing the generic ResNet using 200 images per class (*P* < 0.001, F1 scores, generic = 0.74 ± 0.01, taxonomic = 0.62 ± 0.05, triplet = 0.82 ± 0.01). The MobileNet models using the triplet loss showed good clustering with relatively high Silhouette score (triplet Silhouette score = 0.62 ± 0.05, *k* = 200) and visualization of the clustering (Supplementary Fig. 1) showed similar patterns. Using a ResNet-50 architecture did not result in better classification results when using either taxonomic or triplet loss (Fig. 5). However, silhouette score was better with taxonomic loss when using more than 50 images. The relative poor performance of the taxonomic models might be due to the noise caused by the different vegetation species present in the background of each image.

**Fig. 6:**
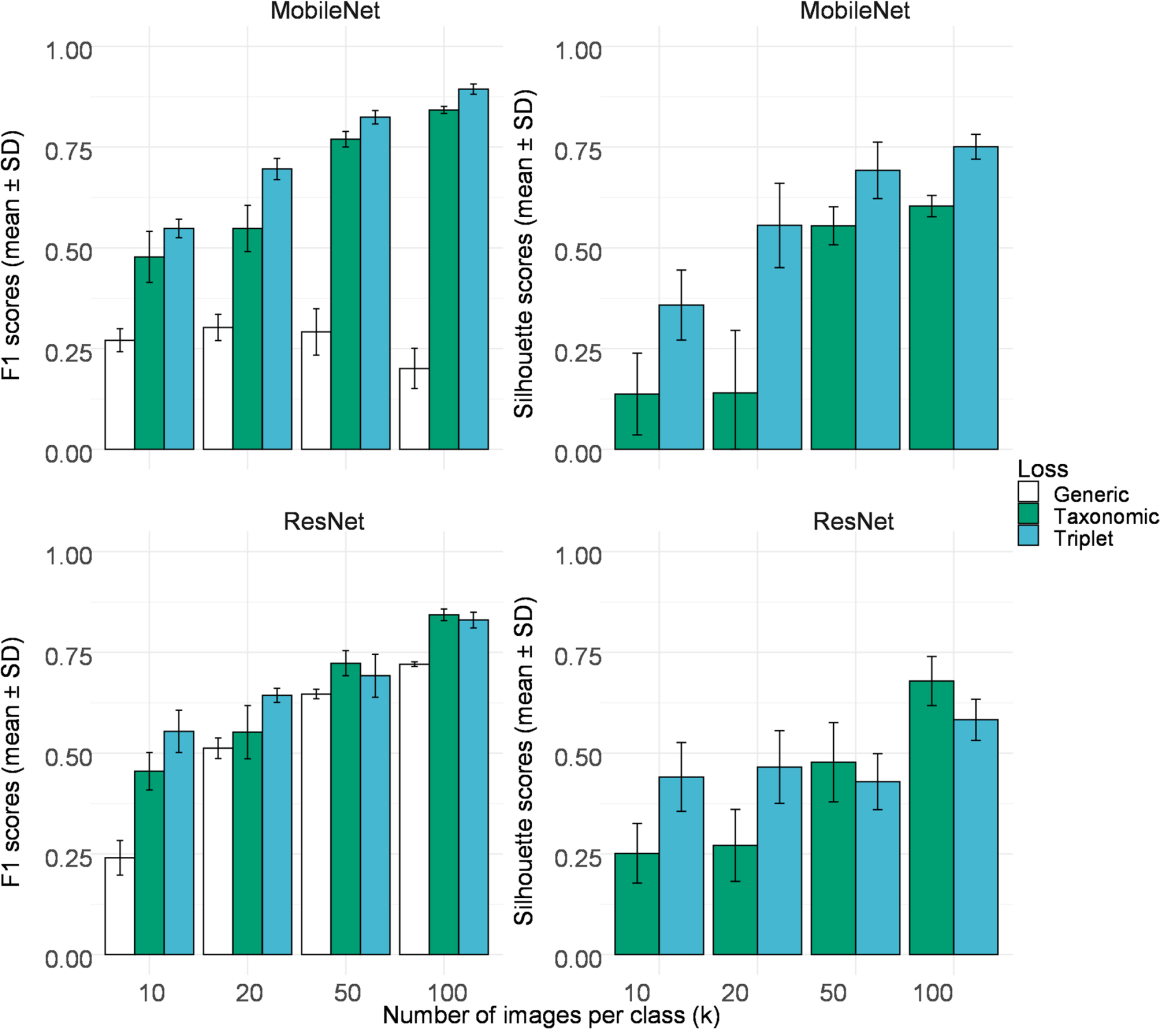
F1 and Silhouette scores classification results for the Plant Seedlings datasets using different loss functions using either MobileNet or ResNet as the base architecture with varying number of images per class (*k*). Simulations were independently carried out (*N* = 10).

In contrast, for the Plant seedlings dataset with its individual weed plants, both taxonomic and triplet loss showed a significant difference between F1 scores for both the MobileNet of ResNet models (*P* < 0.001, Fig. 6). The triplet loss provided the best classification (*k* = 100), when evaluating F1 score (MobileNet Taxonomic loss = 0.85 ± 0.01 vs. Triplet of 0.91 ± 0.01, *P* < 0.001) but not for ResNet (Taxonomic loss = 0.743 ± 0.03 vs. Triplet 0.721 ± 0.047) although this later difference was not significant (*P* > 0.05).

Cluster organization (Supplementary Fig. 2), as evaluated by the Silhouette scores, was better for the triplet loss using the MobileNet architecture, while ResNet favored better clusters using the taxonomical loss (*P* < 0.001 for all models, Fig. 6, k = 100).

### Early growth stages and classification error

It was next verified if the model was able to classify the different weed species present in the WPD dataset but at low BBCH stages (10–12, Table 3). A specific portion of the dataset was selected and MobileNet models were trained on each specific BBCH stages instead of training a global model to recognize each species. A general MobileNet model was also trained as a control.

Globally, taxonomic loss yielded results similar to triplet loss when using the precision score as metrics. At BBCH 10, taxonomic loss showed some improvement, but results were not statistically different (BBCH 10 taxonomic Precision scores 0.75 ± 0.055 vs. triplet 0.72 ± 0.07, *P* > 0.05). Thus, taxonomic loss had classification scores similar to triplet loss when classifying weed seedlings at low BBCH stages using only 20 images for the creation of the models,. However, both loss functions showed better results to the generic loss function.

**Table 3:** Classification of WPD at low BBCH using MobileNet^1^.

Loss^2^	BBCH	Precision	Recall	F1	Silhouette^3^
Generic	10	0.61 ± 0.04	0.62 ± 0.04	0.57 ± 0.04	NA
Taxonomic	10	0.75 ± 0.06	0.67 ± 0.06	0.68 ± 0.05	0.66 ± 0.10
Triplet	10	0.72 ± 0.07	0.70 ± 0.06	0.69 ± 0.06	0.67 ± 0.08
Generic	11	0.65 ± 0.05	0.46 ± 0.09	0.40 ± 0.09	NA
Taxonomic	11	0.91 ± 0.04	0.90 ± 0.04	0.90 ± 0.04	0.74 ± 0.04
Triplet	11	0.91 ± 0.02	0.89 ± 0.03	0.89 ± 0.03	0.72 ± 0.04
Generic	12	0.72 ± 0.04	0.63 ± 0.05	0.59 ± 0.07	NA
Taxonomic	12	0.88 ± 0.02	0.86 ± 0.03	0.86 ± 0.03	0.69 ± 0.06
Triplet	12	0.87 ± 0.03	0.87 ± 0.03	0.87 ± 0.03	0.67 ± 0.06

^1^WPD dataset (BBCH10-12) with *k* = 20 during training. ^2^Generic loss refers to the original model used in classification with standard cross-entropy loss. ^3^Sillhouette scores of the resulting clusters after applying t-SNE to the training set. Simulations were independently carried out (*N* = 10) and results represent the mean ± SD (see also supplementary tables).

**Fig. 7:**
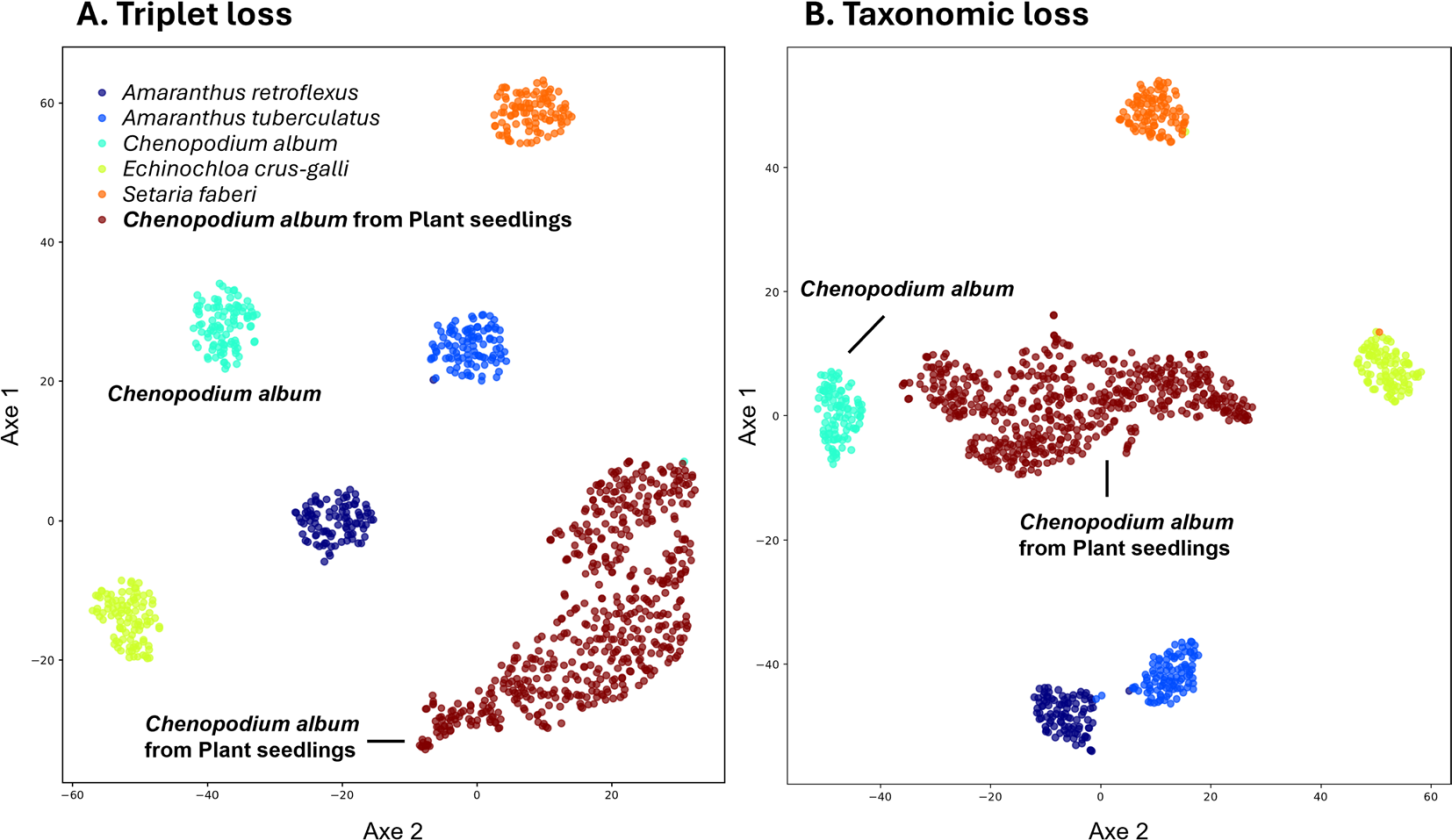
Visualization of the resulting embedding vector using *t*-SNE clustering (*k* = 50, MobileNet) of prediction for new samples using a model trained using the WPD dataset, but predicting on images from the Plant seedling dataset. In (**A**) using the classical triplet loss and in (**B**) using the taxonomic loss.

To further evaluate the taxonomical loss function, classification of 538 *Chenopodium album* from the Plant Seedling datasets was performed using the best MobileNet models trained on the WPD dataset for both triplet and taxonomic loss (Sect. 3.1). As can be observed in Fig. 7A and B, visualization of the final embedding vectors shows that images from the Plant seedlings dataset are organized in their own cluster for both models. This might result from the different background and light conditions found between both datasets. Similarly, since no image augmentation was used, the generated models were not generalized models. However, while the triplet loss clustering (Fig. 7A) resulted in a cluster distant from the WPD *Chenopodium album* embedding, the use of the taxonomical loss embedding resulted in the Plant seedlings images of the same species to be clustered near the WPD *Chenopodium album* class embedding (Fig. 7B). This resulted in 495 images correctly classified for the taxonomic model (with 43 misclassified as *Amaranthus tuberculatus*), while the triplet model classified the majority of images as *Amaranthus tuberculatus* (481) and only 57 as *Chenopodium album*.

## Discussion

The classification of weed seedlings at the species level is important for the robotization of agriculture^52^ and for the overall reduction of herbicides through targeted applications^53^. Previous studies using deep learning to classify weed seedlings, such as the Deepseedlings approach, achieved high weed classification with F1 scores between 0.924 and 0.990^54^, while others obtained lower scores, between 0.79 and 0.90 for red clover (*Trifolium pratense*) and alfalfa (*Medicago sativa*) until the first leave stage^55^. Other studies also showed good performance using different YOLO object detectors for weed detection at various phenological stages^53,56,57^.

Deep learning networks with triplet loss have been shown to perform well for the classification of leaf types and associated diseases, with an accuracy of ~ 90% using 80 leaf images per class^27^. Other networks using different CNN architectures have also generated good classification accuracies (74.33–95.75%) of leaf shapes, using a low number of images of leaves (5–20 per class)^29^.

This project was thus initiated to extend the work of Ge^31^ with the aim of revisiting the HTL loss, applying it to early stage weed seedlings classification from related weed species, but using a low number of annotated images for each class. Different authors have demonstrated that taxonomy-based training schemes can improve classification accuracy when applied to plant images^13^, leaves^29,58^ or flowers^59^. Two different CNN architectures, the MobileNet and the ResNet-50, were chosen for the simulations since both have shown good accuracy for weed classification, even with class imbalance^20,60^. Due to the limited availability of well-annotated weed seedling image datasets, such as the Plant seedlings dataset^23^, with a few notable exceptions^55,61^, a specific weed seedling dataset was created with distinct BBCH stages and with related species difficult to distinguish by weed scientists.

Investigation revealed that HTL loss improved classification accuracy over the general CNN architecture (Sect. 3.1). It was then hypothesized that a fixed taxonomy might be better suited to weed classification. A novel taxonomical loss function, using a fixed taxonomy, was then formulated to take advantage of this a priori information available at the start of the training steps. The overall approach is related to the more recent work of Le and collaborators^13^ that incorporated *species*, *parent-child* and *sibling* relationships into their Adaptive Hierarchical Loss (AHL) loss calculations.

Our evaluation of the novel taxonomical loss showed some improvement in classification over HTL loss (Fig. 3; Sect. 3.1) but model performance was not always superior to the standard triplet loss. For instance, in the original DeepWeeds paper^38^, the authors achieved an average score of > 90% precision for each species using 600 images for each class. Our best model using deep learning networks was the triplet loss using MobileNet (*k =* 600) images with a comparable global precision of 90.9% for this dataset, while the taxonomic loss only achieved 85.6% (Supplementary Tables).

However, training using the Plant seedlings dataset led to F1 scores of 0.85 for taxonomical loss, and 0.91 for triplet loss using 100 images per class for a total of 1,200 images (MobileNet, Sect. 3.2). This is an improvement on the overall F1 score of 0.86 achieved by the authors using 3,961 images^61^. Increasing the number of images to 200 images per class, the taxonomical loss model achieved F1 score of 0.92 (MobileNet model, Supplementary Tables), with a model that could be transferable to embedded devices^22^.

While the performance of the taxonomic loss was not always superior, it resulted in better clustering of the embedding vectors for some models and datasets, as measured by the Silhouette score (Sect. 3.2 and Supplementary Figs. 1 and 2). It also resulted in a better organization of the embedding space following the a priori hierarchy. This improvement in the embedding vector representation was originally why the HTL loss was selected^31^. This may also account for the strong classification of weed images taken from different populations in distinct environments (Sect. 3.3, Fig. 7B), despite the challenge of cross-domain transfer often faced by deep learning networks using few-shot learning (FSL)^62^.

Different techniques could be applied to improve weed seedlings classification using the taxonomic loss. For one, newer CNN architectures such as the MobileOne^63^ or MobileVit-related^64^ could be evaluated to improve the overall taxonomic model accuracy. Second, different plant datasets such as PlantNet-300 K^65^ containing 1,081 species (306,146 color images) exists and could be used to pretrain a specific deep learning network, before applying the FSL training, to further improve classification accuracy.

Another potential research would be to experiment different augmentation techniques that could be applied to the WPD datasets to achieve a better generalization of the classification, such as geometric and color transformations^48^, or more advanced techniques such as AugMix^66^. These augmentation techniques could potentially help in reducing classification errors generated by the presence of different background plant species (e.g. crops) or soil conditions (e.g. wet/dry/rock presence/soil color).

## Conclusion

The application of hierarchical loss models was revisited throughout this study using a known and defined taxonomy to guide the training of a deep learning network using a novel taxonomic loss function. This prior knowledge could be useful when the volume of annotated data is limited, or when classifying information is already available. We demonstrated that both metric learning approaches (triplet and taxonomical) could be used for fine-grained classification under data scarcity. However, the taxonomical loss approach demonstrated superior classification performance when applied to images from a new domain, opening interesting research avenues into new FSL techniques.

## Supplementary Information

Below is the link to the electronic supplementary material.

Supplementary Material 1

## Data Availability

Data and source code could be accessed at github.com/etiennelord/TaxonomicalLoss.
