# Supplementary material for: Taxonomical loss for weed seedlings image classification
Source: Sci Rep. 2026 Jan 24;16:3837. doi: 10.1038/s41598-025-33961-0 (PMC12852093; doi:10.1038/s41598-025-33961-0)
Supplement: Supplementary file 1 — Supplementary Material 1 [file 41598_2025_33961_MOESM1_ESM.pdf]

Supplementary Information

Taxonomical loss for weed seedlings image classification

Hans-Olivier Fontaine[1,2], Samuel Foucher[1], Edith Fallon[2], Marie-Josee Simard[2], and Etienne Lord[2,*]

[1]*Université de Sherbrooke, Department of Applied Geomatics, 2500, boulevard de l'Université, Sherbrooke, QC, Canada, J1K 2R1*
[2]*Agriculture and Agri-Food Canada, St-Jean-sur-Richelieu Research and Development Centre, 430 Gouin Boulevard, Saint-Jean-sur-Richelieu, QC, Canada, J3B 3E6*
Correspondence*:
Etienne.Lord@agr.gc.ca, https://orcid.org/0000-0002-3834-6096

Source code, sample dataset and training scripts can be found at:
github.com/etiennelord/TaxonomicalLoss

Supplementary Figure 1. DeepWeeds

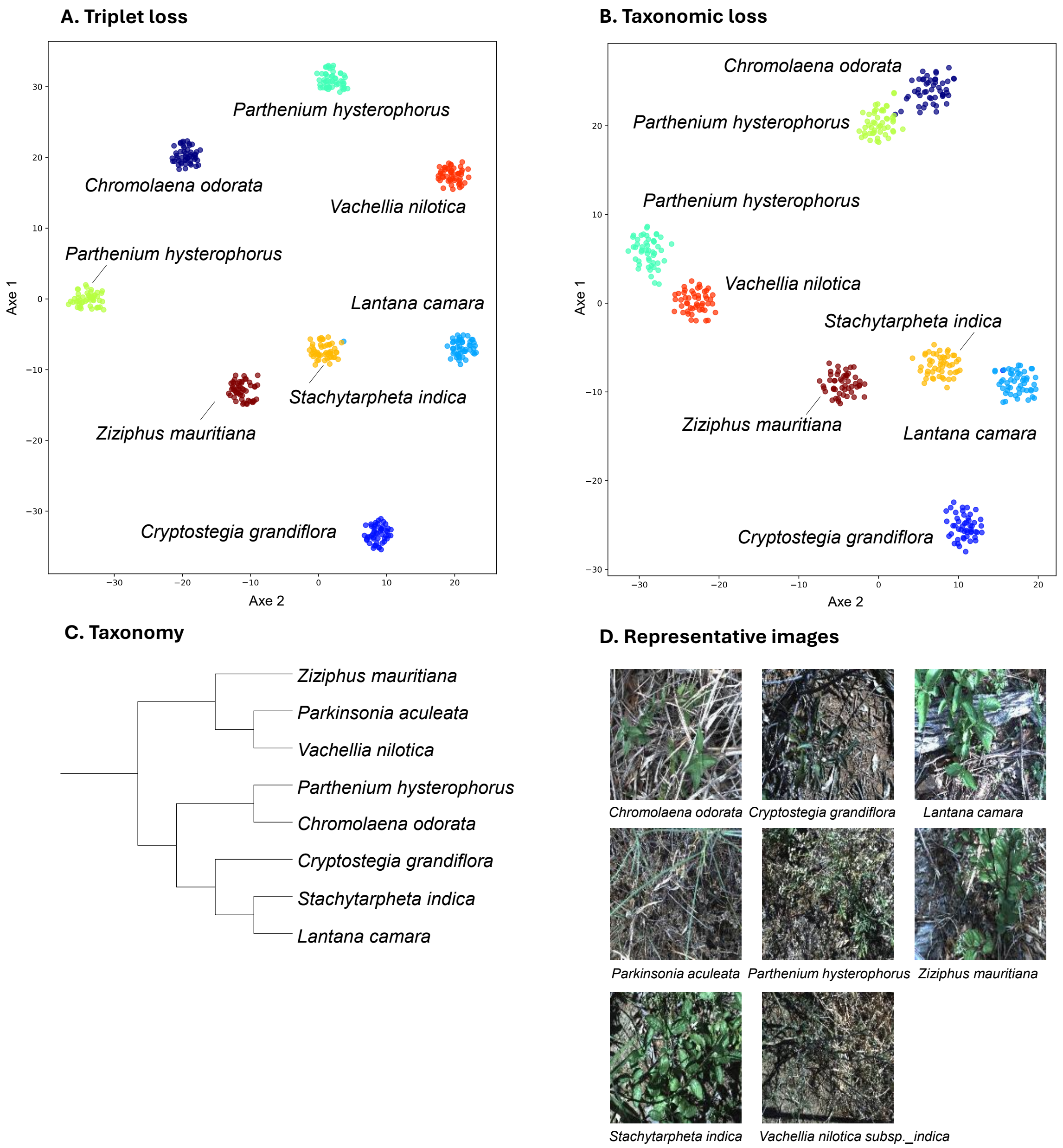

Supplementary Fig. 1. In A and B, Visualization of the DeepWeeds training set embedding vector using *t*-SNE clustering (*k*=50, MobileNet) for each classes after 100 epochs using either (A) triplet loss or (B) taxonomic loss. In C, the fixed taxonomy of the DeepWeeds dataset used during training. In D, representative images from the DeepWeeds dataset [38].

Supplementary Figure 2. Plant Seedlings

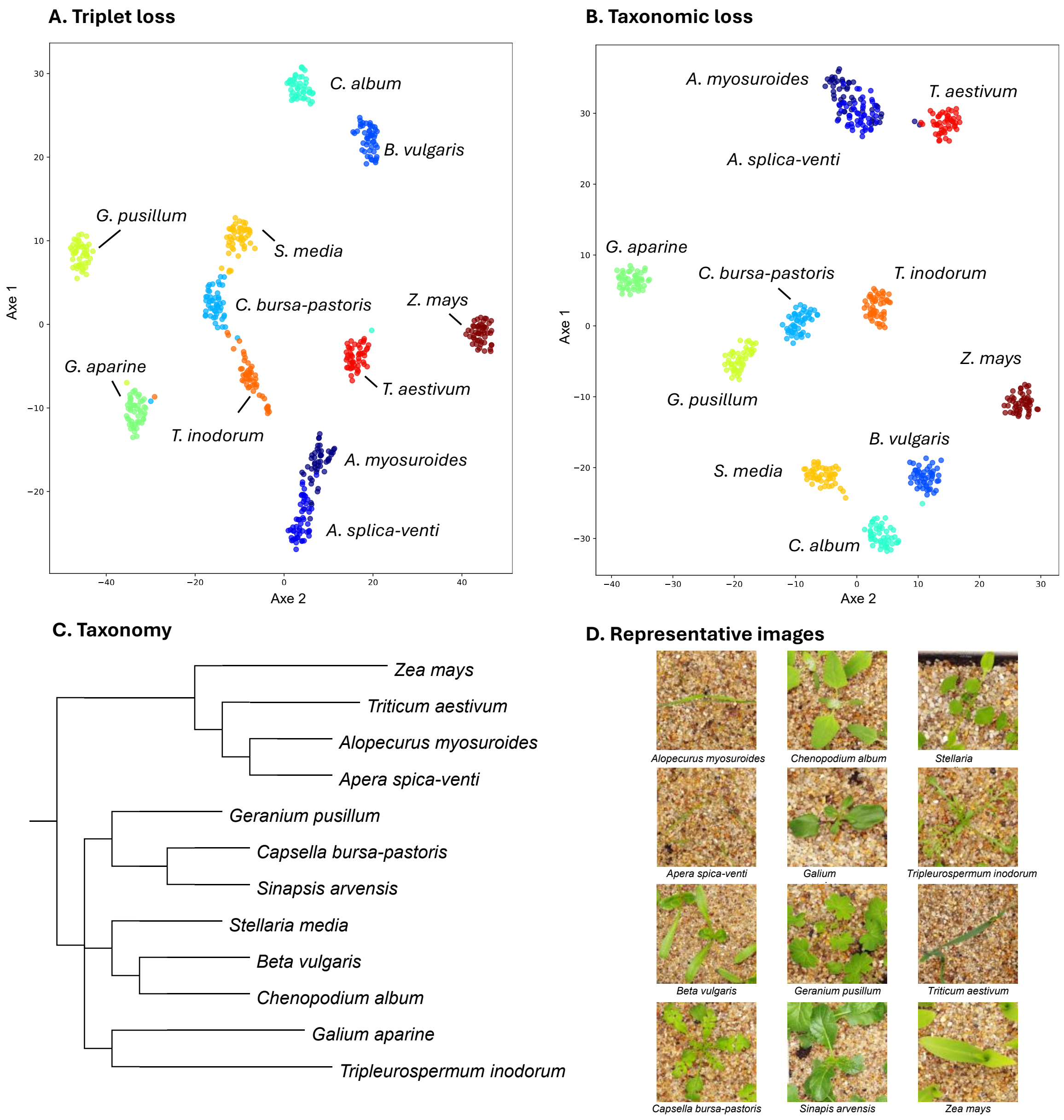

Supplementary Fig. 2. In A and B visualization of the Plant Seedlings training set embedding vector using *t*-SNE clustering (*k*=50, MobileNet) for each classes after 100 epochs using either (A) triplet loss or (B) taxonomic loss. In C, the fixed taxonomy used during training. In D, representative images from the Plant Seedlings dataset [23].

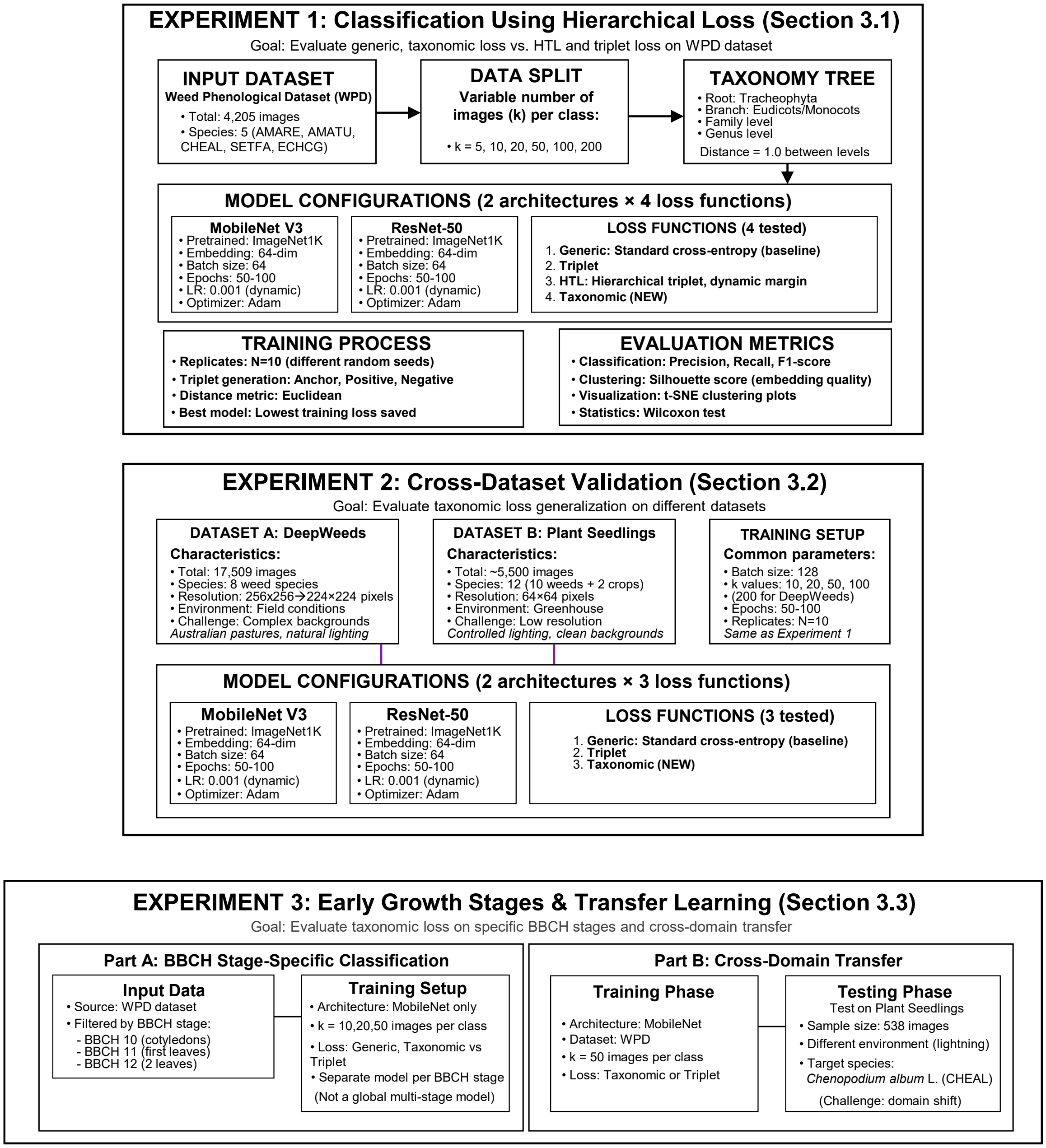

Supplementary Fig. 3. Schematic view of the different experimental procedures and goals for each section.

Supplementary Table 1. Plant image datasets

Dataset Name	Total Images	Total Size	Format	Classes / Species
**WPD**<br><br>*Github* **:** WPDv2.tar.gz<br><br>(fully annotated dataset with identifiable BBCH stages. BBCH10-14 were used in the experiments)	3,920	792.94 MB	JPEG	**5 weed species :**<br>• Amaranthus retroflexus (BBCH09-14): 934<br>• Amaranthus tuberculatus (BBCH09-BBCH21+): 409<br>• Chenopodium album (BBCH09-BBCH21+): 832<br>• Echinochloa crus-galli (BBCH09-BBCH21+): 768<br>• Setaria faberi (BBCH09-BBCH21+): 977<br><br>**BBCH10-14 (3,230 images):**<br>• Amaranthus retroflexus (AMARE): 658<br>• Amaranthus tuberculatus (AMATU): 292<br>• Chenopodium album (CHEAL): 655<br>• Echinochloa crus-galli (ECHCG): 725<br>• Setaria faberi(SETFA): 900
**WPD (BBCH 10-12)**<br><br>*Github* **:** WPD_separated_BBCH.tar.gz	2,467	504.8 MB	JPEG	**5 weed species :**<br>• Amaranthus retroflexus (AMARE): 570<br>• Amaranthus tuberculatus (AMATU): 238<br>• Chenopodium album (CHEAL): 498<br>• Echinochloa crus-galli (ECHCG): 581<br>• Setaria faberi(SETFA): 580
**DeepWeeds subset – resized to 256x256 pixels (Olsen et al. 2019)**	8,403	224.02 MB	JPEG	**8 invasive weed species:**<br>• Chromolaena odorata: 1,074<br>• Cryptostegia grandiflora: 1,009<br>• Lantana camara: 1,063<br>• Parkinsonia aculeata: 1,031<br>• Parthenium hysterophorus: 1,022<br>• Stachytarpheta indica: 1,016<br>• Vachellia nilotica subsp. indica: 1,062<br>• Ziziphus mauritiana: 1,126
**Plant Seedlings subset – resized to 64x64 pixels (Giselsson et al. 2017)**	5,539	41.35 MB	PNG	**12 plant species (weeds & crops):**<br>• Alopecurus myosuroides: 309<br>• Apera spica-venti: 762<br>• Beta vulgaris: 463<br>• Capsella bursa-pastoris: 274<br>• Chenopodium album: 538<br>• Galium aparine: 335<br>• Geranium pusillum: 576<br>• Sinapis arvensis: 452<br>• Stellaria media: 713<br>• Tripleurospermum inodorum: 607<br>• Triticum aestivum: 253<br>• Zea mays: 257

Supplementary results WPD

Statistics for the WPD datasets. Simulations were carried out 10 times for each condition.

N	Model[1]	Loss[2]	k	F1	Precision	Recall	Silhouette[3]
10	mobilenet	generic	5	0.383 ± 0.095	0.32 ± 0.061	0.258 ± 0.061	NA*
10	mobilenet	htl	5	0.433 ± 0.068	0.316 ± 0.065	0.301 ± 0.057	NA*
10	mobilenet	taxonomic	5	0.454 ± 0.044	0.397 ± 0.038	0.385 ± 0.040	0.179 ± 0.008
10	mobilenet	triplet	5	0.417 ± 0.048	0.372 ± 0.042	0.355 ± 0.043	0.175 ± 0.009
10	mobilenet	generic	10	0.394 ± 0.061	0.354 ± 0.037	0.299 ± 0.041	NA*
10	mobilenet	htl	10	0.394 ± 0.041	0.349 ± 0.03	0.343 ± 0.032	NA*
10	mobilenet	taxonomic	10	0.534 ± 0.031	0.484 ± 0.047	0.483 ± 0.044	0.243 ± 0.098
10	mobilenet	triplet	10	0.512 ± 0.052	0.489 ± 0.052	0.486 ± 0.051	0.251 ± 0.110
10	mobilenet	generic	20	0.416 ± 0.049	0.391 ± 0.02	0.323 ± 0.027	NA*
10	mobilenet	htl	20	0.523 ± 0.052	0.448 ± 0.061	0.453 ± 0.057	NA*
10	mobilenet	taxonomic	20	0.584 ± 0.020	0.552 ± 0.026	0.558 ± 0.024	0.413 ± 0.09
10	mobilenet	triplet	20	0.628 ± 0.030	0.59 ± 0.034	0.595 ± 0.031	0.527 ± 0.115
10	mobilenet	generic	50	0.566 ± 0.030	0.463 ± 0.035	0.406 ± 0.067	NA*
10	mobilenet	htl	50	0.535 ± 0.076	0.483 ± 0.067	0.492 ± 0.066	NA*
10	mobilenet	taxonomic	50	0.698 ± 0.028	0.659 ± 0.026	0.668 ± 0.025	0.692 ± 0.039
10	mobilenet	triplet	50	0.778 ± 0.022	0.763 ± 0.019	0.765 ± 0.020	0.794 ± 0.074
10	mobilenet	generic	100	0.624 ± 0.016	0.574 ± 0.023	0.557 ± 0.032	NA*
10	mobilenet	htl	100	0.613 ± 0.065	0.565 ± 0.073	0.578 ± 0.07	NA*
10	mobilenet	taxonomic	100	0.768 ± 0.016	0.745 ± 0.020	0.75 ± 0.018	0.868 ± 0.011
10	mobilenet	triplet	100	0.863 ± 0.008	0.855 ± 0.011	0.856 ± 0.010	0.837 ± 0.026
10	mobilenet	generic	200	0.668 ± 0.010	0.658 ± 0.011	0.659 ± 0.011	NA*
10	mobilenet	htl	200	0.720 ± 0.037	0.673 ± 0.036	0.685 ± 0.034	NA*
10	mobilenet	taxonomic	200	0.866 ± 0.011	0.851 ± 0.012	0.854 ± 0.012	0.839 ± 0.004
10	mobilenet	triplet	200	0.927 ± 0.010	0.924 ± 0.010	0.925 ± 0.010	0.787 ± 0.008
10	resnet	generic	5	0.430 ± 0.031	0.404 ± 0.031	0.405 ± 0.03	NA*
10	resnet	taxonomic	5	0.287 ± 0.051	0.251 ± 0.051	0.250 ± 0.053	0.146 ± 0.351
10	resnet	triplet	5	0.407 ± 0.088	0.316 ± 0.071	0.315 ± 0.075	0.178 ± 0.203
10	resnet	generic	10	0.487 ± 0.043	0.466 ± 0.041	0.468 ± 0.043	NA*
10	resnet	taxonomic	10	0.304 ± 0.037	0.289 ± 0.032	0.282 ± 0.033	0.184 ± 0.159
10	resnet	triplet	10	0.404 ± 0.057	0.359 ± 0.048	0.351 ± 0.055	0.208 ± 0.122
10	resnet	generic	20	0.551 ± 0.020	0.532 ± 0.021	0.535 ± 0.021	NA*
10	resnet	taxonomic	20	0.401 ± 0.068	0.365 ± 0.067	0.373 ± 0.069	0.075 ± 0.090
10	resnet	triplet	20	0.323 ± 0.067	0.305 ± 0.059	0.309 ± 0.061	0.079 ± 0.051
10	resnet	generic	50	0.634 ± 0.008	0.621 ± 0.012	0.624 ± 0.011	NA*
10	resnet	taxonomic	50	0.513 ± 0.091	0.474 ± 0.088	0.477 ± 0.088	0.546 ± 0.059
10	resnet	triplet	50	0.574 ± 0.084	0.523 ± 0.068	0.535 ± 0.071	0.344 ± 0.163
10	resnet	generic	100	0.698 ± 0.018	0.686 ± 0.017	0.689 ± 0.017	NA*
10	resnet	taxonomic	100	0.718 ± 0.037	0.682 ± 0.043	0.687 ± 0.042	0.812 ± 0.080
10	resnet	triplet	100	0.546 ± 0.124	0.517 ± 0.119	0.525 ± 0.121	0.215 ± 0.228
10	resnet	generic	200	0.755 ± 0.013	0.744 ± 0.012	0.747 ± 0.012	NA*
10	resnet	taxonomic	200	0.848 ± 0.029	0.833 ± 0.032	0.835 ± 0.031	0.797 ± 0.008
10	resnet	triplet	200	0.599 ± 0.140	0.569 ± 0.137	0.579 ± 0.139	0.192 ± 0.149

* Not evaluated. [1]MobileNetV3 with ImageNet1k_V2 pretrained weights and ResNet50 with InageNet_V2 pretrained weights. [2]Generic loss refers to the original model used in classification, while HTL, Taxonomic and Triplet loss refers to a network with one specific loss function. [3]Silhouette scores of the resulting clusters after applying t-SNE to the training set.

Statistics for the WPD datasets. Simulation were carried out 10 times for each condition.

Pairwise comparisons using Wilcoxon rank sum exact test with Bonferroni P value adjustment method.

	**WPD mobilenet F1-score *P-value***			
**k**		**generic**	**htl**	**taxonomic**
**5**	**htl**	0.286		
	**taxonomic**	6.49e-05	**0.008**	
	**triplet**	**0.002**	0.086	0.286
**10**	**htl**	**0.046**		
	**taxonomic**	**6.49e-05**	**6.49e-05**	
	**triplet**	**6.49e-05**	**6.49e-05**	0.853
**20**	**htl**	**0.0001**		
	**taxonomic**	**6.49e-05**	**0.0001**	
	**triplet**	**6.49e-05**	**8.66e-05**	**0.0089**
**50**	**htl**	**0.0357**		
	**taxonomic**	**4.33e-05**	**3.4e-05**	
	**triplet**	**4.33e-05**	**3.4e-05**	**4.33e-05**
**100**	**htl**	0.279		
	**taxonomic**	**6.49e-05**	**6.49e-05**	
	**triplet**	**6.49e-05**	**6.49e-05**	**6.49e-05**
**200**	**htl**	**0.029**		
	**taxonomic**	**6.49e-05**	**6.49e-05**	
	**triplet**	**6.49e-05**	**6.49e-05**	**6.49e-05**

	**WPD ResNet-50 F1-score (*P-value*)**		
**k**		**generic**	**taxonomic**
**5**	**taxonomic**	**3.24e-05**	
	**triplet**	**0.010**	**0.043**
**10**	**taxonomic**	**3.24e-05**	
	**triplet**	**0.0004**	**0.0038**
**20**	**taxonomic**	**3.24e-05**	
	**triplet**	**3.24e-05**	0.0524
**50**	**taxonomic**	**0.00038**	
	**triplet**	**0.0014**	0.105
**100**	**taxonomic**	0.578	
	**triplet**	**6.49e-05**	**0.00025**
**200**	**taxonomic**	**3.24e-05**	
	**triplet**	**0.0001**	**3.24e-05**

Pairwise comparisons using Wilcoxon rank sum exact test with Bonferroni P value adjustment method.

	**MobileNet silhouette (vs triplet)**
**k**	**taxonomic (*P-value*)**
**5**	0.168
**10**	0.795
**20**	**0.0015**
**50**	**0.005**
**100**	**0.0004**
**200**	**3.24e-05**

	**ResNet-50 silhouette (vs triplet)**
**k**	**taxonomic (*P-value*)**
**5**	NA
**10**	0.578
**20**	**0.0015**
**50**	**0.075**
**100**	**1.08e-05**
**200**	**1.08e-05**

Supplementary results DeepWeeds

Statistics for the DeepWeeds datasets. Simulations were carried out 10 times for each condition.

N	Model[1]	Loss[2]	k	F1	Precision	Recall	Silhouette[3]
10	mobilenet	generic	5	0.446 ± 0.040	0.382 ± 0.022	0.353 ± 0.030	NA*
10	mobilenet	taxonomic	5	0.383 ± 0.046	0.323 ± 0.026	0.302 ± 0.036	0.061 ± 0.053
10	mobilenet	triplet	5	0.393 ± 0.053	0.341 ± 0.031	0.327 ± 0.036	0.033 ± 0.080
10	mobilenet	generic	10	0.474 ± 0.024	0.415 ± 0.028	0.388 ± 0.035	NA*
10	mobilenet	taxonomic	10	0.423 ± 0.036	0.384 ± 0.026	0.380 ± 0.03	0.168 ± 0.079
10	mobilenet	triplet	10	0.474 ± 0.034	0.434 ± 0.031	0.428 ± 0.031	0.199 ± 0.080
10	mobilenet	generic	20	0.572 ± 0.021	0.526 ± 0.018	0.515 ± 0.020	NA*
10	mobilenet	taxonomic	20	0.428 ± 0.024	0.411 ± 0.019	0.409 ± 0.018	0.237 ± 0.043
10	mobilenet	triplet	20	0.533 ± 0.029	0.511 ± 0.026	0.507 ± 0.028	0.338 ± 0.068
10	mobilenet	generic	50	0.668 ± 0.013	0.656 ± 0.015	0.654 ± 0.015	NA*
10	mobilenet	taxonomic	50	0.487 ± 0.018	0.477 ± 0.015	0.477 ± 0.016	0.211 ± 0.053
10	mobilenet	triplet	50	0.622 ± 0.035	0.611 ± 0.035	0.609 ± 0.034	0.388 ± 0.095
10	mobilenet	generic	100	0.711 ± 0.009	0.712 ± 0.009	0.710 ± 0.010	NA*
10	mobilenet	taxonomic	100	0.568 ± 0.014	0.559 ± 0.016	0.560 ± 0.015	0.279 ± 0.028
10	mobilenet	triplet	100	0.725 ± 0.027	0.719 ± 0.027	0.720 ± 0.027	0.506 ± 0.075
10	mobilenet	generic	200	0.737 ± 0.008	0.739 ± 0.008	0.737 ± 0.008	NA*
10	mobilenet	taxonomic	200	0.623 ± 0.048	0.620 ± 0.047	0.620 ± 0.047	0.310 ± 0.060
10	mobilenet	triplet	200	0.814 ± 0.014	0.812 ± 0.013	0.812 ± 0.013	0.617 ± 0.045
10	mobilenet	taxonomic	600	0.851 ± 0.027	0.856 ± 0.027	0.851 ± 0.027	0.588 ± 0.036
10	mobilenet	triplet	600	0.891 ± 0.013	0.909 ± 0.013	0.908 ± 0.082	0.647 ± 0.012
10	resnet	generic	5	0.531 ± 0.028	0.530 ± 0.022	0.524 ± 0.025	NA*
10	resnet	taxonomic	5	0.285 ± 0.030	0.273 ± 0.03	0.259 ± 0.029	-0.032 ± 0.106
10	resnet	triplet	5	0.326 ± 0.050	0.276 ± 0.037	0.264 ± 0.039	0.063 ± 0.048
10	resnet	generic	10	0.594 ± 0.015	0.592 ± 0.014	0.588 ± 0.013	NA*
10	resnet	taxonomic	10	0.343 ± 0.042	0.332 ± 0.039	0.329 ± 0.041	0.062 ± 0.083
10	resnet	triplet	10	0.415 ± 0.029	0.380 ± 0.030	0.378 ± 0.031	0.217 ± 0.076
10	resnet	generic	20	0.659 ± 0.018	0.660 ± 0.018	0.655 ± 0.018	NA*
10	resnet	taxonomic	20	0.371 ± 0.033	0.359 ± 0.030	0.36 ± 0.031	0.011 ± 0.096
10	resnet	triplet	20	0.388 ± 0.032	0.373 ± 0.030	0.372 ± 0.03	0.098 ± 0.060
10	resnet	generic	50	0.725 ± 0.013	0.726 ± 0.012	0.723 ± 0.013	NA*
10	resnet	taxonomic	50	0.456 ± 0.024	0.440 ± 0.024	0.442 ± 0.024	0.150 ± 0.045
10	resnet	triplet	50	0.392 ± 0.021	0.384 ± 0.018	0.385 ± 0.019	0.034 ± 0.039
10	resnet	generic	100	0.779 ± 0.005	0.780 ± 0.005	0.779 ± 0.005	NA*
10	resnet	taxonomic	100	0.572 ± 0.041	0.568 ± 0.042	0.566 ± 0.043	0.373 ± 0.054
10	resnet	triplet	100	0.568 ± 0.023	0.555 ± 0.026	0.559 ± 0.024	0.288 ± 0.040
10	resnet	generic	200	0.812 ± 0.007	0.811 ± 0.007	0.812 ± 0.007	NA*
10	resnet	taxonomic	200	0.699 ± 0.026	0.692 ± 0.026	0.692 ± 0.027	0.517 ± 0.042
10	resnet	triplet	200	0.717 ± 0.019	0.706 ± 0.021	0.709 ± 0.02	0.420 ± 0.046

* Not evaluated [1]MobileNetV3 with ImageNet1k_V2 pretrained weights and ResNet50 with ImageNet_V2 pretrained weights [2]Generic loss refers to the original model used in classification with standard cross-

entropy loss, while Taxonomic and Triplet loss refers to a network with either loss function. [3]Silhouette scores of the resulting clusters after applying t-SNE to the training set.

Statistics for the Deep Weeds datasets. Simulations were carried out 10 times for each condition.

Pairwise comparisons using Wilcoxon rank sum exact test with Bonferroni P value adjustment method.

	DeepWeeds mobilenet F1-score (*P-value*)		
**k**		**generic**	**taxonomic**
**5**	**taxonomic**	**0.011**	
	**triplet**	0.246	0.246
**10**	**taxonomic**	0.739	
	**triplet**	**0.022**	**0.008**
**20**	**taxonomic**	**3.24e-05**	
	**triplet**	0.481	**3.24e-05**
**50**	**taxonomic**	**3.24e-05**	
	**triplet**	**0.0003**	**3.24e-05**
**100**	**taxonomic**	**3.24e-05**	
	**triplet**	0.165	**3.24e-05**
**200**	**taxonomic**	**3.24e-05**	N/A
	**triplet**	**3.24e-05**	**3.24e-05**

	DeepWeeds ResNet-50 F1-score (*P-value*)		
**k**		**generic**	**taxonomic**
**5**	**taxonomic**	**3.24e-05**	
	**triplet**	**3.24e-05**	0.684
**10**	**taxonomic**	**3.24e-05**	
	**triplet**	**3.24e-05**	**0.006**
**20**	**taxonomic**	**3.24e-05**	
	**triplet**	**3.24e-05**	0.393
**50**	**taxonomic**	**3.24e-05**	
	**triplet**	**3.24e-05**	**0.0001**
**100**	**taxonomic**	**3.24e-05**	
	**triplet**	**3.24e-05**	0.853
**200**	**taxonomic**	**3.24e-05**	
	**triplet**	**3.24e-05**	0.123

Pairwise comparisons using Wilcoxon rank sum exact test with Bonferroni P value adjustment method.

	**Mobilenet silhouette (vs triplet)**
**k**	**taxonomic (*P-value*)**
**5**	0.739
**10**	0.528
**20**	**0.0038**
**50**	**0.0032**
**100**	**0.108e-05**
**200**	**0.108e-05**

	**ResNet-50 silhouette (vs triplet)**
**k**	**taxonomic (*P-value*)**
**5**	**0.0288**
**10**	**0.001**
**20**	**0.0288**
**50**	**4.33e-5**
**100**	**0.0015**
**200**	**0.0002**

Supplementary results PlantSeedlings

Statistics for the PlantSeedlings datasets. Simulations were carried out 10 times for each condition.

N	Model[1]	Loss[2]	k	F1	Precision	Recall	Silhouette[3]
10	mobilenet	generic	5	0.341 ± 0.049	0.239 ± 0.039	0.216 ± 0.039	NA*
10	mobilenet	taxonomic	5	0.361 ± 0.054	0.319 ± 0.034	0.323 ± 0.038	0.004 ± 0.126
10	mobilenet	triplet	5	0.462 ± 0.061	0.394 ± 0.059	0.399 ± 0.056	0.146 ± 0.113
10	mobilenet	generic	10	0.387 ± 0.041	0.294 ± 0.035	0.271 ± 0.029	NA*
10	mobilenet	taxonomic	10	0.509 ± 0.057	0.47 ± 0.065	0.477 ± 0.063	0.137 ± 0.101
10	mobilenet	triplet	10	0.597 ± 0.038	0.537 ± 0.026	0.548 ± 0.023	0.358 ± 0.087
10	mobilenet	generic	20	0.438 ± 0.038	0.316 ± 0.042	0.303 ± 0.033	NA*
10	mobilenet	taxonomic	20	0.573 ± 0.061	0.542 ± 0.056	0.548 ± 0.057	0.14 ± 0.155
10	mobilenet	triplet	20	0.734 ± 0.029	0.685 ± 0.027	0.696 ± 0.026	0.556 ± 0.104
10	mobilenet	generic	50	0.485 ± 0.029	0.298 ± 0.058	0.291 ± 0.058	NA*
10	mobilenet	taxonomic	50	0.781 ± 0.019	0.765 ± 0.02	0.769 ± 0.019	0.555 ± 0.047
10	mobilenet	triplet	50	0.846 ± 0.015	0.817 ± 0.018	0.824 ± 0.016	0.692 ± 0.07
10	mobilenet	generic	100	0.456 ± 0.049	0.218 ± 0.047	0.201 ± 0.050	NA*
10	mobilenet	taxonomic	100	0.852 ± 0.009	0.838 ± 0.009	0.842 ± 0.009	0.603 ± 0.027
10	mobilenet	triplet	100	0.910 ± 0.011	0.889 ± 0.013	0.894 ± 0.013	0.751 ± 0.031
10	mobilenet	generic	200	0.782 ± 0.012	0.736 ± 0.013	0.751 ± 0.012	NA*
10	mobilenet	taxonomic	200	0.918 ± 0.006	0.899 ± 0.005	0.906 ± 0.005	0.690 ± 0.009
10	mobilenet	triplet	200	0.950 ± 0.005	0.931 ± 0.009	0.936 ± 0.008	0.765 ± 0.026
10	resnet	generic	5	0.348 ± 0.031	0.241 ± 0.04	0.207 ± 0.044	NA*
10	resnet	taxonomic	5	0.359 ± 0.056	0.314 ± 0.047	0.315 ± 0.051	0.077 ± 0.066
10	resnet	triplet	5	0.467 ± 0.038	0.388 ± 0.043	0.394 ± 0.044	0.168 ± 0.084
10	resnet	generic	10	0.412 ± 0.038	0.268 ± 0.045	0.24 ± 0.043	NA*
10	resnet	taxonomic	10	0.500 ± 0.048	0.445 ± 0.046	0.455 ± 0.046	0.251 ± 0.074
10	resnet	triplet	10	0.602 ± 0.046	0.545 ± 0.053	0.554 ± 0.052	0.441 ± 0.085
10	resnet	generic	20	0.538 ± 0.022	0.508 ± 0.026	0.512 ± 0.025	NA*
10	resnet	taxonomic	20	0.585 ± 0.063	0.544 ± 0.067	0.552 ± 0.066	0.271 ± 0.089
10	resnet	triplet	20	0.680 ± 0.023	0.636 ± 0.019	0.643 ± 0.017	0.466 ± 0.09
10	resnet	generic	50	0.666 ± 0.012	0.642 ± 0.012	0.647 ± 0.012	NA*
10	resnet	taxonomic	50	0.743 ± 0.03	0.716 ± 0.032	0.723 ± 0.031	0.477 ± 0.098
10	resnet	triplet	50	0.721 ± 0.047	0.681 ± 0.056	0.692 ± 0.053	0.429 ± 0.07
10	resnet	generic	100	0.743 ± 0.005	0.714 ± 0.006	0.721 ± 0.006	NA*
10	resnet	taxonomic	100	0.855 ± 0.014	0.839 ± 0.015	0.843 ± 0.015	0.679 ± 0.061
10	resnet	triplet	100	0.852 ± 0.016	0.823 ± 0.021	0.830 ± 0.020	0.583 ± 0.051

* Not evaluated [1]MobileNetV3 with ImageNet1k_V2 pretrained weights and ResNet50 with ImageNet_V2 pretrained weights [2]Generic loss refers to the original model used in classification with standard cross-entropy loss, while Taxonomic and Triplet loss refers to a network with either loss function. [3]Silhouette scores of the resulting clusters after applying t-SNE to the training set.

Statistics for the Plant Seedlings datasets. Simulations were carried out 10 times for each condition.

Pairwise comparisons using Wilcoxon rank sum exact test with Bonferroni P value adjustment method.

	**PlantSeedling mobilenet F1-score (*P-value*)**		
**k**		**generic**	**taxonomic**
**5**	**taxonomic**	**6.49e-05**	
	**triplet**	**6.49e-05**	**0.005**
**10**	**taxonomic**	**3.24e-05**	
	**triplet**	**3.24e-05**	**0.003**
**20**	**taxonomic**	**3.24e-05**	
	**triplet**	**3.24e-05**	**3.24e-05**
**50**	**taxonomic**	**3.24e-05**	
	**triplet**	**3.24e-05**	**3.24e-05**
**100**	**taxonomic**	**3.24e-05**	
	**triplet**	**3.24e-05**	**3.24e-05**
**200**	**taxonomic**	**3.24e-05**	
	**triplet**	**3.24e-05**	**3.24e-05**

	**PlantSeedlingResNet-50 F1-score (*P-value*)**		
**k**		**generic**	**taxonomic**
**5**	**taxonomic**	0.0004	
	**triplet**	3.24e-05	**0.0015**
**10**	**taxonomic**	**3.24e-05**	
	**triplet**	**3.24e-05**	**0.0007**
**20**	**taxonomic**	0.143	
	**triplet**	**3.24e-05**	**0.003**
**50**	**taxonomic**	**3.24e-05**	
	**triplet**	0.126	0.190
**100**	**taxonomic**	**3.24e-05**	
	**triplet**	0.126	0.190

Pairwise comparisons using Wilcoxon rank sum exact test with Bonferroni P value adjustment method.

	**Mobilenet silhouette (vs triplet)**
**k**	**taxonomic (*P-value*)**
**5**	0.10
**10**	**2.16e-5**
**20**	**2.16e-5**
**50**	**0.0003**
**100**	**1.08e-05**
**200**	**1.08e-05**

	**ResNet-50 silhouette (vs triplet)**
**k**	**taxonomic (*P-value*)**
**5**	**0.0112**
**10**	**0.0003**
**20**	**0.0003**
**50**	0.3930
**100**	**0.0010**

Supplementary results to table 3 – WPD classification at different BBCH stages

Model[1]	Loss	BBCH	k	Precision	Recall	F1	Silhouette[3]
mobilenet	generic	BBCH10	10	0.484 ± 0.060	0.446 ± 0.078	0.3720± 0.080	N/A*
mobilenet	taxonomic	BBCH10	10	0.677 ± 0.050	0.556 ± 0.076	0.578 ± 0.075	0.656 ± 0.172
mobilenet	triplet	BBCH10	10	0.647 ± 0.058	0.570 ± 0.034	0.580 ± 0.038	0.459 ± 0.174
mobilenet	generic	BBCH11	10	0.465 ± 0.118	0.339 ± 0.097	0.261 ± 0.119	NA*
mobilenet	taxonomic	BBCH11	10	0.809 ± 0.067	0.776 ± 0.081	0.781 ± 0.080	0.669 ± 0.158
mobilenet	triplet	BBCH11	10	0.793 ± 0.061	0.771 ± 0.060	0.770 ± 0.065	0.804 ± 0.126
mobilenet	generic	BBCH12	10	0.570 ± 0.097	0.484 ± 0.071	0.391 ± 0.086	NA*
mobilenet	taxonomic	BBCH12	10	0.746 ± 0.042	0.707 ± 0.041	0.712 ± 0.047	0.583 ± 0.116
mobilenet	triplet	BBCH12	10	0.789 ± 0.046	0.758 ± 0.060	0.760 ± 0.057	0.632 ± 0.193
mobilenet	generic	BBCH10	20	0.614 ± 0.036	0.617 ± 0.041	0.574 ± 0.042	NA*
mobilenet	taxonomic	BBCH10	20	0.748 ± 0.055	0.663 ± 0.058	0.684 ± 0.050	0.661 ± 0.104
mobilenet	triplet	BBCH10	20	0.721 ± 0.067	0.694 ± 0.061	0.695 ± 0.064	0.697 ± 0.075
mobilenet	generic	BBCH11	20	0.651 ± 0.046	0.458 ± 0.090	0.402 ± 0.089	NA*
mobilenet	taxonomic	BBCH11	20	0.911 ± 0.039	0.901 ± 0.039	0.903 ± 0.038	0.735 ± 0.044
mobilenet	triplet	BBCH11	20	0.910 ± 0.020	0.898 ± 0.032	0.899 ± 0.031	0.722 ± 0.039
mobilenet	generic	BBCH12	20	0.719 ± 0.036	0.630 ± 0.051	0.587 ± 0.071	NA*
mobilenet	taxonomic	BBCH12	20	0.875 ± 0.024	0.859 ± 0.029	0.863 ± 0.027	0.685 ± 0.060
mobilenet	triplet	BBCH12	20	0.874 ± 0.030	0.867 ± 0.033	0.868 ± 0.033	0.671 ± 0.059
mobilenet	generic	BBCH10	50	0.774 ± 0.026	0.726 ± 0.019	0.735 ± 0.020	NA*
mobilenet	taxonomic	BBCH10	50	0.836 ± 0.029	0.777 ± 0.044	0.795 ± 0.036	0.625 ± 0.033
mobilenet	triplet	BBCH10	50	0.858 ± 0.046	0.822 ± 0.048	0.833 ± 0.045	0.473 ± 0.099
mobilenet	generic	BBCH11	50	0.843 ± 0.027	0.825 ± 0.027	0.825 ± 0.025	NA*
mobilenet	taxonomic	BBCH11	50	0.970 ± 0.012	0.967 ± 0.014	0.967 ± 0.014	0.631 ± 0.029
mobilenet	triplet	BBCH11	50	0.976 ± 0.012	0.975 ± 0.013	0.975 ± 0.013	0.524 ± 0.080
mobilenet	generic	BBCH12	50	0.859 ± 0.018	0.836 ± 0.019	0.840 ± 0.019	NA*
mobilenet	taxonomic	BBCH12	50	0.945 ± 0.013	0.941 ± 0.016	0.942 ± 0.015	0.621 ± 0.035
mobilenet	triplet	BBCH12	50	0.962 ± 0.013	0.960 ± 0.013	0.960 ± 0.013	0.521 ± 0.062

* Not evaluated. [1]MobileNetV3 with ImageNet1k_V2 pretrained weights. [2]Generic loss refers to the original model used in classification with standard cross-entropy loss, while Taxonomic and Triplet loss refers to a network with either loss function. [3]Silhouette scores of the resulting clusters after applying *t*-SNE to the training set. Simulations were independently carried out ten times (*N*=10) and we report the average ± SD.
